# Targeting cardiovascular immunometabolism with phytomedicines: an emerging therapeutic technique

**DOI:** 10.3389/fcvm.2026.1941655

**Published:** 2026-10-29

**Authors:** Acharya Balkrishna, Shalini Mishra, Maneesha Rana, Vedpriya Arya, Shalini Singh

**Affiliations:** 1 Patanjali Herbal Research Department, Patanjali Research Foundation, Haridwar, India; 2 Department of Pharmaceutical Sciences, Gurukula Kangri (Deemed to be University), Haridwar, India

**Keywords:** cardiovascular disease, immunometabolism, inflammation, macrophage polarization, mitochondrial dysfunction, phytomedicine

## Abstract

Cardiovascular diseases (CVDs) continue to be leading causes of morbidity and death globally, with chronic inflammation contributing to disease progression and residual cardiovascular risk despite breakthroughs in conventional treatment. Immunometabolism, defined as the bidirectional connection of cellular metabolism and immune-cell activity, is an emerging approach for understanding the relationship between metabolic failure and cardiovascular inflammation. Several regulatory pathways, such as AMPK, mTOR, NLRP3, SIRT1, and NRF2, have been linked to metabolic and inflammatory responses in CVD. This review critically assesses the molecular evidence relating these pathways to CVD and investigates the potential for specific phytochemicals and phytomedicines to modify cardiovascular immunometabolism. Curcumin, resveratrol, berberine, quercetin, and andrographolide have all been shown to have pathway-specific effects in cellular and animal models of inflammation, oxidative stress, mitochondrial function, macrophage responses, and endothelial dysfunction. However, mechanistic activity should not be confused with demonstrated cardiovascular therapeutic effectiveness. Poor bioavailability, phytochemical variability, insufficient standardisation, pharmacokinetic uncertainty, possible herb-drug interactions, and a lack of sufficiently powered randomised clinical studies continue to impede translation. Future research should focus on standardised formulations, mechanistic validation in relevant cardiovascular models, pharmacokinetic and safety characterisation, and well-designed clinical trials combining immunometabolic biomarkers. The integration of multi-omics, systems pharmacology, nanophytomedicine, and computational techniques may help to advance evidence-based phytopharmaceutical development.

## 1 Introduction

Cardiovascular diseases (CVDs), including coronary artery disease, stroke, heart failure, and peripheral vascular disease, continue to be the greatest causes of morbidity and mortality across the globe, causing approximately a third of total fatalities globally (1). Although significant progress has been made in the pharmaceutical treatment options of lipid-lowering drugs, antihypertensives, antiplatelets, and sodium-glucose cotransporter-2 (SGLT2) inhibitors, the incidence of CVDs is increasing globally due to aging, metabolic conditions, and lifestyle risks (2). There are several individuals at risk of experiencing recurrent cardiovascular events even after reaching the target goals of therapy due to the presence of residual inflammation. Low-grade chronic inflammation plays an important role in endothelial dysfunction, plaque progression, heart muscle damage, and adverse cardiac remodelling, thus demonstrating the importance of anti-inflammatory therapy along with standard methods of treatment (3). Immunometabolism refers to the bidirectional interplay between cellular and immune cell activity. In this interaction, metabolic states control immune-cell activation, differentiation, survival, and effector activities, whereas immunological and inflammatory signals alter cellular metabolic programs. This relationship in CVD relates metabolic problems such as obesity, insulin resistance, hyperglycemia, dyslipidaemia, and oxidative stress to inflammatory responses involving macrophages, T cells, endothelial cells, and cardiomyocytes. In addition, immunometabolism refers to the regulation of the activation, differentiation, and activities of immune cells via metabolic pathways, whereas inflammatory chemicals change the cellular metabolic program (4). Obesity, diabetes, dyslipidaemia, high blood pressure, and oxidative stress all generate in macrophages, T lymphocytes, endothelial cells, and cardiomyocytes that contribute to CVD. The dysregulation of the signalling pathways such as AMP-activated protein kinase (AMPK), mechanistic target of rapamycin (mTOR), nuclear factor-*κ*B (NF-*κ*B), NLRP3 inflammasome, Sirtuin-1 (SIRT1), and nuclear factor erythroid 2-related factor 2 (NRF2) creates a link between metabolic disorder and cardiovascular inflammation, thus, making immunometabolism a potential therapeutic target (5). Phytomedicines represent one of the promising therapies being considered for treating diseases based on their capability of regulating various linked molecular pathways. These drugs contain bioactive agents like polyphenols, flavonoids, alkaloids, and terpenoids and possess anti-inflammatory, antioxidant, immunomodulating, and metabolic regulation activities through a series of modulation of multiple signalling pathways (6). This multicomponent therapy is consistent with the systems biological theory that considers CVD a complicated disorder resulting from the interaction between different pathways including immune, metabolic, oxidative, and vascular pathways. However, there must be a rational phytopharmaceutical development of phytomedicines supported by a series of studies including formulation, pharmacokinetics, mechanism validation, and clinical trials (7, 8). In contrast to previous reviews that are primarily concentrated either on cardiovascular inflammation or on individual phytochemicals, the present review article highlights the integration of recent developments in cardiovascular immunometabolism with multitarget regulation using phytomedicine. The special attention is paid to metabolic reprogramming of immune cells, mitochondrial quality control, trained immunity, translation problems, and perspectives of precision phytomedicine in the future.

## 2 Literature search strategy

A structured literature search was carried out using PubMed, Web of Science, and Google Scholar to find material related to cardiovascular immunometabolism and the possible involvement of phytomedicines and phytochemicals in modifying immunometabolic pathways linked with CVD. The search covers publications published between January 2013 and June 2026. Search terms included “cardiovascular disease”, “cardiovascular inflammation”, “immunometabolism”, “atherosclerosis”, “macrophage metabolism”, “AMPK”, “mTOR”, “NF-*κ*B”, “NLRP3 inflammasome”, “SIRT1”, “NRF2”, “phytomedicine”, “phytochemicals”, “natural products”, “curcumin”, “resveratrol”, “berberine”, “quercetin”, and “cardiovascular inflammation”. Studies that addressed at least one of the following were considered eligible: (i) mechanisms of cardiovascular immunometabolism; (ii) metabolic regulation of immune or vascular cells relevant to CVD; (iii) experimental cardiovascular effects of phytochemicals or medicinal plants; or (iv) translational or clinical evidence relevant to cardiovascular applications. Based on their applicability to the goals of the mini-review, experimental research, clinical studies, systematic or narrative reviews, and mechanistic studies were taken into consideration. Research unrelated to the chosen phytochemicals or medicinal plants, studies without a clear cardiovascular or immunometabolic relationship, and publications lacking mechanistic or translational significance were also excluded. After screening titles and abstracts for relevance, the full text was evaluated as needed. Recent mechanistic studies, cardiovascular experimental models, clinical studies, and reviews addressing translational limitations were given particular emphasis. The search was intended to identify and critically evaluate representative and mechanistically relevant articles rather than to produce a comprehensive systematic evidence synthesis because this article is a narrative mini review rather than a systematic review.

## 3 Cardiovascular immunometabolism: molecular basis and pathophysiological significance

Immunometabolism represents the interface of cell metabolism and immune function, in which metabolic routes dictate activation, differentiation, cytokine secretion, cell survival and effector function of immune cells, rather than simply generating energy for them. Cellular metabolism has now come to be accepted as a primary control of immune cell identity, function and inflammation (9, 10). Under quiet conditions, immune cells mainly use mitochondrial oxidative phosphorylation (OXPHOS) and fatty acid oxidation (FAO) to satisfy their basic energy needs which maintain immune functions and tissue balance. This metabolic program is typical of naïve T cells, regulatory T cells, tissue-resident macrophages, and dendritic cells (4, 11). Immune cells undergo metabolic reprogramming during inflammatory and infectious processes such as the promotion of glycolysis and the Warburg effect in cancer cells (12). Although glycolysis produces less ATP than OXPHOS, it metabolic intermediates needed for the synthesis of nucleotides, amino acids, and lipids required to produce pro-inflammatory cytokines and reactive oxygen species (ROS) by macrophages, neutrophils, dendritic cells and T lymphocytes (5). Alternatively activated macrophages (M2), regulatory T cells (Tregs), and memory T cells remain mainly oxidative and use FAO to promote tolerance, tissue regeneration, and inflammatory resolution (13, 14). The pro-inflammatory immune responses are mainly fuelled by glycolysis, whereas immune homeostasis, tissue repair and anti-inflammatory functions are performed by mitochondrial OXPHOS and FAO (5). The pentose phosphate pathway is the source for the substrates required for the biosynthesis of nucleotides (ribose-5-phosphate). In addition, NADPH that is provided by PPP can be used as a substrate for ROS production by NADPH oxidase or can also be involved in cell redox state balance maintenance. However, hyperactivation of the pentose phosphate pathway could induce oxidative stress and subsequent endothelial dysfunction in atherosclerosis (15).

Metabolism of lipids and amino acids plays an important role in immune responses. Lipid metabolism regulates immune activation via its effects on cholesterol homeostatic mechanisms and on the assembly of lipid rafts and thereby controls inflammatory signalling (16). Regulation of amino acid metabolism plays an equally crucial role in modulating immune system activation. Glutamine serves as a major anaplerosis of intermediates of the TCA cycle during immune activation; metabolism of the amino acid arginine affects macrophage polarisation through regulation of inducible nitric oxide synthase (iNOS) and arginase (Arg) activity, and tryptophan metabolism regulates immune tolerance in T cells through differentiation via the indoleamine 2,3-dioxygenase (IDO) pathway (17). Several metabolites have signalling activities beyond their classical metabolic functions and directly affect immune responses. Examples of metabolites that can be responsible for the regulation of TF function, accessibility of chromatin and inflammatory gene expression to link cellular metabolism with immune homeostasis are succinate, citrate, fumarate and itaconate (18). In the meantime, quality control for mitochondria via mitophagy also plays a critical role in regulating immune homeostasis. Dysfunctional mitochondria, accumulation of dysfunctional mitochondria, increased mtDNA leakage and mitochondrial ROS and inflammatory signalling with NLRP3 inflammasome activation (19). Nutrient- and energy-sensing signalling cascades tightly orchestrate immunometabolic reprogramming. mTOR enhances anabolism, glycolysis, protein synthesis, and pro-inflammatory immune responses during nutritional surplus, and AMPK, as an energy sensor, enhances FAO, mitochondrial biogenesis, and autophagy and inhibits inflammatory signalling pathways (20). Although cellular context, dietary availability, inflammatory stimuli, and tissue microenvironment all affect immune cell metabolic and functional characteristics, the balance between AMPK- and mTOR-associated signalling plays a role. Other factors are also involved to fine-tune this response. HIF-1*α* promotes glycolysis and inflammatory cytokines under hypoxia, while SIRT1 inhibits NF-*κ*B signalling and decreases ROS (21). The activation of the NLRP3 inflammasome promotes interleukin-1β and interleukin-18 leading to sterile inflammation (22). Recent evidence suggests that metabolic reprogramming may continue after acute immune activation and contribute to trained immunity via prolonged metabolic and epigenetic remodelling (23).

## 4 Metabolic reprogramming of immune cells in CVD

Immunometabolic reprogramming is a key connection between metabolic diseases and chronic inflammation in CVD. Regular cardiometabolic stress, such as obesity, insulin resistance, hyperglycaemia, dyslipidaemia, hypertension and oxidative stress, changes glucose, lipid, amino acid and mitochondrial metabolism in immune and vascular cells, which causes endothelial dysfunction, atherosclerosis, myocardial remodelling and heart failure (24, 25). Recent advances in single-cell transcriptomics and spatial multi-omics have revealed significant metabolic heterogeneity within immune-cell populations, implicating cell-specific metabolic pathways as potential targets for therapy. Macrophages exhibit remarkable metabolic plasticity throughout the progression of CVD (26). Metabolic reprogramming including alteration in glycolysis, loss of Tricarboxylic acid (TCA) cycle function and increase of immunometabolites (succinate, citrate and fumarate) caused by oxLDL in atheromatous plaques cause activation of hypoxia-inducible factor-1*α* (HIF-1*α*), NF-*κ*B, and the NLRP3 inflammasome which sustain inflammatory cytokine production (25). Concomitant mitochondrial dysfunction, ROS production, impaired cholesterol efflux, and defective mitophagy contribute to foam-cell formation and plaque progression. However, inflammation-resolving macrophages promote efferocytosis, tissue repair and stabilization of plaques through preserved mitochondrial metabolism and effective cholesterol handling.

Chronic hypercholesterolaemia induces trained immunity resulting in persistent macrophage activation. Similarly, the adaptive immune response regulation by metabolic rewiring, pro-inflammatory Th1 and Th17 cells are abundant in glycolytic settings; in contrast, T regulatory (Treg) cells depend on oxidative metabolism for inducing immune tolerance, as dysregulated metabolism interferes with their suppressive capacity and induces vascular inflammation (27). Treg stability is suppressed by mTOR signalling dysregulation and impaired mitochondria are associated with exacerbation of vascular inflammation. Regulatory B cells use an oxidative metabolic program for sustaining their anti-inflammatory functions. Moreover, vascular and innate immune cell types undergo a metabolic reprogramming (28). Metabolic dysfunction of endothelial cells suffering from disrupted shear stress and metabolic damage is characterized by impaired mitochondria, loss of uncoupling eNOS and generation of oxidative stress and endothelial activation (29). The process of metabolic remodelling takes place in smooth muscle cells, which results in phenotypic shift and vascular calcification, while the cardiac fibroblast cells increase their rate of glycolysis and glutaminolysis for myocardial fibrosis. The dendritic cells increase their glycolysis process to activate antigen presentation. The neutrophils increase the immunothrombosis process by regulating the formation of the neutrophil extracellular traps (NETs) through metabolic regulation. Later, the release of the mitochondrial DNA from the damaged cells activates the cGAS-STING signalling pathway (30, 31).

## 5 Phytomedicines as multitarget regulators of cardiovascular immunometabolism

More recently, growing consciousness of immunometabolism as a central player in cardiovascular homeostasis has led to changing therapeutic targets from the traditional lipid-lowering and antihypertensive mechanisms towards the manipulation of immune cell metabolism (32). It regulates macrophage polarisation, T-cell differentiation, inflammasome activation, mitochondrial functions and endothelial functions modulating the onset and progression of atherosclerosis, MI, HF or hypertension (25, 33). The synergy of its highly concentrated mixture of phenolics, flavonoids, alkaloids, terpenoids and other complex bioactive compounds makes phytomedicines potential modulators of several targets that regulate both metabolic and inflammatory responses (7, 34). The plant-derived phytochemicals regulate cardiovascular immunometabolism to establish equilibrium primarily through the coordination of AMPK, mTOR, NF-*κ*B, NLRP3 inflammasome, SIRT1, and NRF2 signalling pathways as shown in Figure 1. Among them, AMPK plays a crucial role in linking cell energy supply with inflammation signalling pathway acting as a central metabolic controller, reduced activity leads to increased glycolytic energy production, endothelial malfunction, oxidant generation and activation of macrophage-dependent inflammation and on the contrary, elevated AMPK leads to shift of immune response toward anti-inflammatory state.

**Figure 1:**
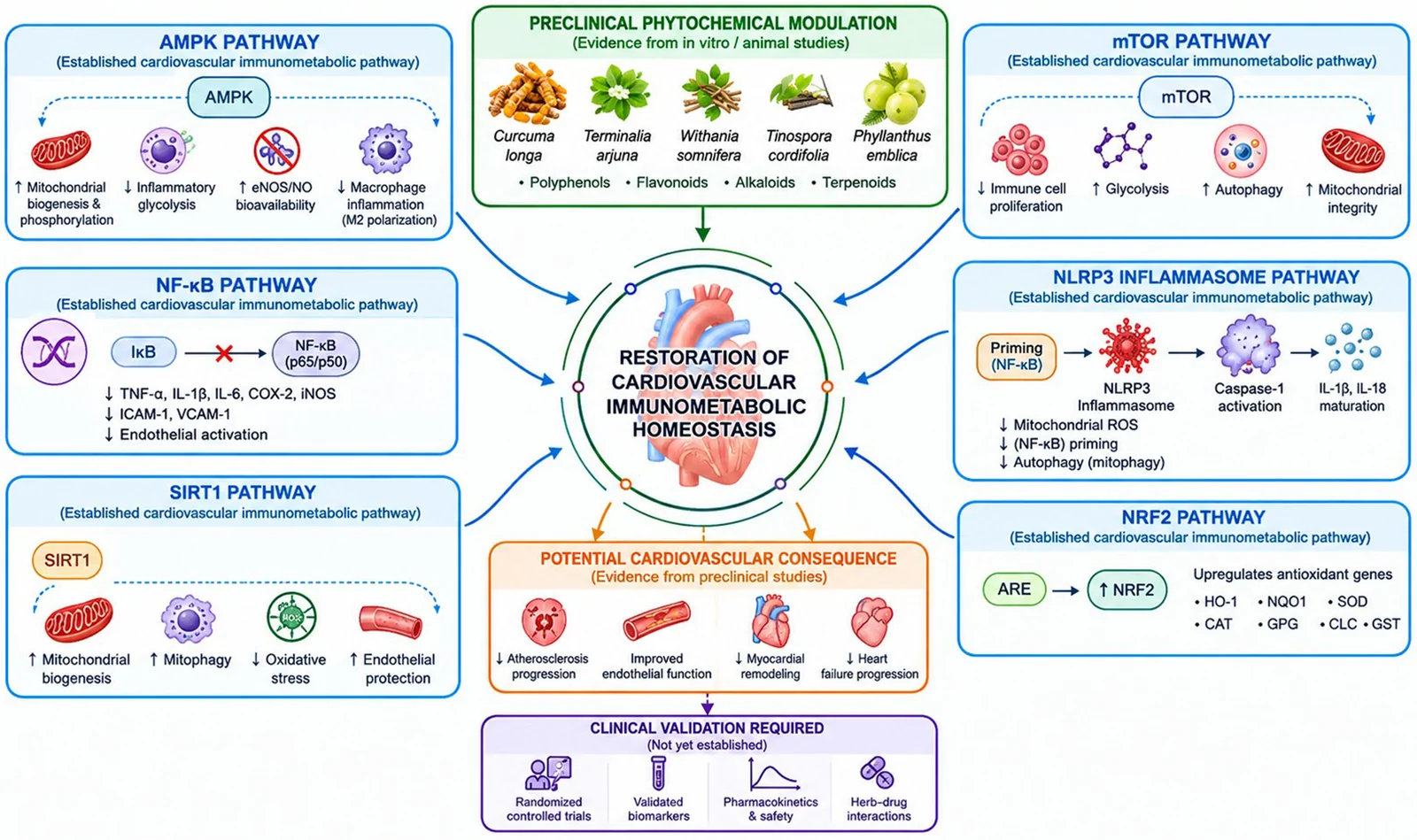
Infographic of cardiovascular immunometabolic pathways and their proposed modulation by phytochemicals. The figure shows 6 interconnected pathways: AMP-activated protein kinase (AMPK), mechanistic target of rapamycin (mTOR), nuclear factor-*κ*B (NF-*κ*B), NLRP3 inflammasome, sirtuin 1 (SIRT1) and nuclear factor erythroid 2-related factor 2 (NRF2). Plant-derived compounds are connected with potential influences on cell metabolism, inflammation, oxidative stress, mitochondrial function, and immune-cell activity. The schematic summarises biologically relevant interactions supported by experimental evidence and proposed mechanisms relevant to cardiovascular disease. Confirmed biological links are indicated by solid arrows, hypothesised or predominantly preclinical interactions are indicated by dashed arrows. Clinical cardiovascular benefits need further validation.

Berberine, curcumin, and resveratrol have been shown in studies to have AMPK-associated effects, such as modulating mitochondrial metabolism and inflammatory signalling. However, these findings are mostly from cellular and animal models, and the extent to which such pathway modification contributes to clinically significant cardiovascular outcomes remains uncertain (8, 35, 36). AMPK activation is a mechanistically plausible pathway through which certain phytochemicals may influence cardiovascular immunometabolism; however, the extent to which AMPK activation directly mediates cardiovascular benefits must be validated in disease-relevant experimental models and clinical studies. Another key immunometabolic regulator is mTOR which controls immune-cell proliferation, glycolytic metabolism, protein synthesis and autophagy. Chronic inflammation, impaired autophagic flux, endothelial dysfunction and adverse cardiac remodelling are all, in part, a result of persistent activation of mTOR, and mTOR accelerates the progression of CVD (4, 37). Experimental studies have shown that the use of such phytochemicals, such as curcumin, ginsenosides and EGCG, have blocked mTOR signal hyperactivity, while also bringing AMPK activities back to normal levels and stimulating the process to promote the generation of autophagy, help the preservation of healthy mitochondria, decrease oxidation and reduce inflammation around the heart (8). The AMPK-mTOR signalling in conditions of CVD has been investigated to address the therapeutic potential of targeting metabolic dysfunction (38). NF-*κ*B, a key signalling hub that links metabolic stress to the activation of inflammatory responses. Increased reactive oxygen species, dyslipidemia, mitochondrial dysfunction and increased glucose levels are known to activate NF-*κ*B, leading to the induction of TNF-alpha, IL-1, IL-6, COX-2 and iNOS. This eventually causes injury of the endothelium and plaque disruption (39). Some phytochemicals such as curcumin, quercetin, luteolin and apigenin have anti-inflammatory effects and break this self-perpetuating cycle of chronic vascular inflammation by suppressing NF-*κ*B and the subsequent inflammatory molecules downstream (40, 41). Emerging evidence also implicated the NLRP3 inflammasome as a key immunometabolic regulator of CVD. Cholesterol crystals, oxidized LDL, mitochondrial ROS and danger associated molecular pattern activated NLRP3 result in maturation of IL-1, IL-18, pyroptosis and subsequent expansion of vascular inflammation (42, 43).

Andrographolide, curcumin, resveratrol, and baicalein can affect NLRP3 inflammasome-associated signalling, primarily through effects on mitochondrial oxidative stress, NF-*κ*B priming, and autophagic processes. However, the cardiovascular relevance and therapeutic significance of these effects yet to be determined (44). Moreover, the AMPK-dependent inactivation of NLRP3 inflammasome provides a critical molecular link between the cellular energy metabolism and the regulation of innate immunity and there is accumulating evidence to support this notion (45). The immunometabolic activity of phytomedicines is also based on their capacity to sustain mitochondrial homeostasis. Mitochondrial dysfunction results in increased ROS production, impaired ATP production and pro-inflammatory activation of immune cells. Resveratrol, quercetin, sulforaphane and curcumin activate SIRT1 and NRF2 to stimulate mitochondrial biogenesis, antioxidant defences and mitophagy, and reduce oxidative stress injury to endothelial cells (46). All these coordinated events facilitate a shift from an immune, pro-inflammatory phenotype to a reparative one. These findings provide a molecular foundation for examining whether modification of mitochondrial quality control influences vascular homeostasis and inflammation-associated cardiac remodelling (47). Conventional systems of medicines have already provided an extensive supply of effective multi-target immune metabolic modulators in the form of medicinal plants such as *T. arjuna*, *C. longa*, *W. somnifera*, *T. cordifolia*, *B. diffusa* and *P. emblica* which provide numerous bioactive phytochemicals targeting simultaneously oxidative stress, inflammatory signalling pathways (NF-*κ*B, NLRP3 inflammasome), mitochondrial functioning (AMPK, SIRT1) and immune cells' metabolic pathways (NRF2) (8). These phytomedicine seems to act on pleiotropic effects within interdependent immunometabolic networks, which explains their possible use as an adjuvant therapy for the prevention and management of CVD. Despite this, further mechanistic studies and prospective clinical trials are required to validate their utility, standardize their preparation, and translate their potential into evidence-based cardiovascular therapeutics (36). The poor oral bioavailability, diverse phytochemical content and non-standardized preparation of phytomedicines, along with the lack of large-scale randomized trials, limit their translation into the clinical practice, despite extensive preclinical validation. There is a need for better integration of systems and network pharmacology with the emerging area of nanophytomedicine and multi-omics to develop standardized phytopharmaceuticals that could target specific cardiovascular immunometabolic nodes and thus aid in their transition towards precision cardiovascular medicine. Although substantial experimental evidence exists about the immunometabolic regulatory actions of many phytochemicals, there is wide variability regarding the level of evidence that exists. Most of the existing data comes from *in vitro* studies and animals, while solid randomized clinical trials are few and far between. Therefore, the existing evidence makes phytochemicals more of an exciting alternative to conventional medicines rather than a replacement.

## 6 Translational advances and emerging therapeutic opportunities

The immune cell metabolism has become a major therapeutic target in response to advances in cardiovascular immunometabolism, which has revolutionized the paradigm of management by providing a non-conventional target to reduce the development and progression of CVD. Current conventional therapies for CVD such as statins, antihypertensives and antithrombotic agents target dyslipidaemia, high blood pressure or thrombosis, respectively, but an increasing amount of clinical evidence suggests several approved drugs are also potent immunometabolic modulators. In addition to the above, phytomedicines offer a unique approach for the complementary modulation of immune-metabolic homeostasis and alleviation of cardiovascular inflammation. Of all approved drugs, metformin is the most studied for its effects on the immune system (2, 3, 48). Other actions of metformin besides lowering glucose include activation of AMPK, inhibition of NF-*κ*B mediated inflammation signalling, improved mitochondrial (OXPHOS) and polarisation of macrophages to the anti-inflammatory M2 phenotype. This effect might be useful to minimize endothelial damage and oxidation/inflammation on the vessel wall and may protect healthy individuals from heart diseases (49). Similarly, rapamycin, a mTOR inhibitor, stimulates autophagy may decrease pathological ventricular hypertrophy and remodelling; nevertheless, chronic immunosuppression and metabolic restrictions remain relevant issues for its broader cardiovascular applicability (50, 51). Targeting the inflammation cascade by inhibiting IL-1 through canakinumab in the CANTOS trial provided convincing clinical data regarding reduction of recurrent cardiovascular events independent of cholesterol lowering, targeting the concept of “residual inflammation” as a therapeutic approach in atherosclerosis (1). At the same time, SGLT2 inhibitors, first designed for the management of diabetes, became highly effective cardiovascular protective agents. SGLT2 inhibitors are an important clinical example of a therapy with cardiovascular benefits that could modify metabolic and inflammatory pathways. Proposed mechanisms include influences on mitochondrial bioenergetics, oxidative stress, and inflammasome-associated signalling, but the proportionate contribution of these processes to their observed cardiovascular repercussions is uncertain (52). However, these results demonstrate that the alteration of immunometabolic pathways is a current fact and this supports the rationale for the development of future multi-targeted approaches.

Unlike conventional drugs, whose function is to target one molecule or pathway, phytochemicals possess the characteristic of polypharmacology since they control several interconnected immunometabolic pathways associated with CVDs. This implies that, it might be more useful to classify plant constituents not according to their botanical origin but based on their targets, to clarify the mode of actions of phytomedicines (6). AMPK activators, including berberine, curcumin and resveratrol, up regulate mitochondrial oxidative metabolism, down regulate inflammatory glycolysis, increase availability of endothelial nitric oxide and prevent macrophage mediated inflammation (53, 54). Both andrographolide and curcumin predominantly inhibit the activation of the NLRP3 inflammasome through the suppression of mitochondrial ROS production, restoration of autophagic flux and reduction of NF-*κ*B priming (55). Flavonoids, typically luteolin and apigenin, largely inhibit NF-*κ*B transcription of pro-inflammatory cytokines and adhesion molecules contributing to vascular inflammation (56). Quercetin and resveratrol stimulate SIRT1 signalling to enhance mitochondrial quality, sulforaphane and curcumin activate the NRF2 mediated antioxidants to strengthen the intrinsic antioxidant defense pathways to diminish oxidative stress (57). The rationale of proposing traditional monotherapy as adjunctive immunometabolic modifiers is their unique ability to influence both pathways simultaneously. Other treatments are traditional plants with complex phytochemicals that can interfere with several signalling pathways involved in inflammatory cardiovascular and metabolic diseases (6, 58). Selected example of species with their predominant bioactive ingredients, primary immunometabolic targets, cardiovascular evidence, and level of scientific evidence in the laboratory (*in vitro* and animal models), and clinical context, rather than listing each botanical, as summarised in Table 1. Moreover, this comparison underscores the magnitude of mechanism-of-action studies and gaps in the scientific literature, and their translational challenges to routine cardiovascular care. One of the major obstacles for the clinical translation of phytomedicines are the low water solubility, poor oral bioavailability, rapid metabolism and tissue accumulation. The newly developing concept of nano-phytomedicine has the potential to break through barriers. Nano-phytomedicine may potentially overcome some of the formulation and pharmacokinetic constraints of poorly soluble phytochemicals. Selective nanocarrier systems have been shown in experimental studies to increase physicochemical stability, apparent bioavailability, cellular exposure, and controlled administration. It must be shown if these formulation improvements translate into better cardiovascular outcomes, increased safety, or clinically significant therapeutic effect.

**Table 1:** Selected phytochemicals modulate cardiovascular immunometabolic pathways: mechanistic and translational evidence.

Phytochemical	Major source	Immunometabolic target/pathway	Experimental cardiovascular evidence	Human/clinical evidence	Evidence level	Major translational limitation	Relevant references
Curcumin	*Curcuma longa*	AMPK, mTOR, NF-*κ*B, NLRP3, NRF2 and SIRT1-related inflammatory, metabolic and oxidative-stress pathways	*In vitro* and animal studies indicate modulation of inflammatory, oxidative-stress, metabolic and endothelial pathways relevant to cardiovascular injury and cardiometabolic dysfunction.	Human studies have evaluated cardiometabolic risk factors; evidence for definitive cardiovascular outcomes remains limited.	Preclinical + human cardiometabolic evidence	Low bioavailability, rapid metabolism and variable systemic exposure	(8, 19, 36, 55)
Resveratrol	*Vitis vinifera* and other plant sources	SIRT1, AMPK, NF-κB and oxidative/inflammatory signaling	Experimental studies indicate modulation of inflammatory, oxidative and metabolic pathways relevant to vascular and cardiovascular dysfunction.	Human studies have evaluated metabolic, inflammatory and vascular/cardiometabolic endpoints; definitive cardiovascular outcome evidence remains limited.	Preclinical + human cardiometabolic evidence	Poor bioavailability, rapid metabolism and pharmacokinetic variability	(8, 36, 40, 54)
Berberine	*Berberis* spp. and other medicinal plants	AMPK and metabolic signaling, with effects on inflammatory and lipid-regulatory pathways	Experimental studies demonstrate modulation of glucose/lipid metabolism, inflammation and cardiovascular-related metabolic pathways.	Human clinical evidence is available for selected cardiometabolic risk factors, including glucose and lipid parameters; definitive cardiovascular outcome efficacy remains insufficiently established.	Preclinical + human cardiometabolic evidence	Variable bioavailability, pharmacokinetic limitations and potential drug interactions	(8, 19, 36)
Quercetin	Various fruits, vegetables and medicinal plants	NF-κB, NRF2, SIRT1 and oxidative/inflammatory signaling	*In vitro* and animal studies indicate modulation of oxidative stress, inflammation, endothelial function and metabolic pathways relevant to cardiovascular disease.	Human studies have evaluated selected vascular and cardiometabolic endpoints; evidence for definitive cardiovascular outcomes remains limited.	Preclinical + limited human cardiometabolic/vascular evidence	Low and variable bioavailability and extensive metabolism	(8, 35, 40, 56)
Andrographolide	*Andrographis paniculata*	NLRP3, NF-κB and inflammatory signaling	Experimental evidence indicates modulation of inflammatory pathways, including NLRP3/NF-κB-related signaling, relevant to cardiovascular inflammation.	No convincing CVD clinical evidence identified from the supplied references.	Preclinical	Insufficient cardiovascular clinical validation and limited translational evidence	(8, 55)
Baicalein	*Scutellaria baicalensis*	NLRP3 and inflammatory/oxidative-stress pathways	Experimental evidence indicates modulation of inflammatory and oxidative pathways relevant to cardiovascular injury.	Limited direct human cardiovascular evidence identified from the supplied references.	Preclinical	Limited clinical validation and uncertain translation of experimental findings	(8, 55)
Ginsenosides	*Panax* spp.	AMPK, mTOR, oxidative-stress, inflammatory and endothelial signaling	Experimental studies indicate modulation of metabolic, inflammatory, oxidative and vascular pathways associated with cardiovascular dysfunction.	Human evidence for selected cardiometabolic/vascular endpoints remains limited and heterogeneous; definitive cardiovascular outcome evidence is insufficient.	Preclinical + limited human evidence	Structural heterogeneity, variable composition, bioavailability and limited standardized clinical evidence	(8, 19, 35)

AMPK, AMP-activated protein kinase; mTOR, mechanistic target of rapamycin; NF-κB, nuclear factor-κB; NLRP3, nucleotide-binding oligomerization domain-like receptor family pyrin domain-containing 3; NRF2, nuclear factor erythroid 2-related factor 2; SIRT1, sirtuin-1.

Furthermore, many hurdles remain for phytomedicine-based immunometabolic therapies in the clinical cardiovascular arena. Current problems include variations in phytochemical profiles, poor standard extraction methods, insufficient pharmacokinetic profiles, the possible herb-drug interactions and insufficient well powered randomized controlled clinical trials. The integration of systems pharmacology, network pharmacology, multi-omics technology, and AI-assisted phytochemical discovery is expected to help recognize phytomedicines with clearly defined molecular targets and consistent therapeutic efficacy, which may complement contemporary immunometabolic therapies and progress toward personalized cardiovascular medicine. Whilst many of the reported molecular mechanisms such as AMPK activation, macrophage polarisation, NLRP3 inflammasome inhibition, and mitochondrial restoration have been shown mostly under experimental conditions. The clinical significance, therapeutic dosage, safety, and reproducibility need to be validated by randomized controlled trials prior to any cardiovascular applications.

## 7 Discussion

Cardiovascular immunometabolism offers an emerging concept for comprehending the bidirectional link between cellular metabolism and immune function in CVD. Chronic vascular inflammation, endothelial dysfunction, atherogenesis, myocardial remodelling, and heart failure have all been linked to metabolic reprogramming in macrophages, T cells, dendritic cells, endothelial cells, neutrophils, and cardiomyocytes (4, 5, 24). AMPK, mTOR, NF-*κ*B, HIF-1*α*, NLRP3 inflammasome, SIRT1, and NRF2 are possible immunometabolic targets. These pathways, however, should be seen as mechanistically relevant targets rather than proven CVD treatment solutions (5, 33). The data presented in this review suggests that certain phytochemicals, such as curcumin, resveratrol, berberine, quercetin, andrographolide, baicalein, and ginsenosides, might influence interrelated immunometabolic pathways in experimental systems. Berberine, curcumin, and resveratrol, for example, have been linked to AMPK-mediated metabolic and inflammatory effects, whereas curcumin, ginsenosides, and epigallocatechin gallate have been linked to mTOR signalling and autophagy (8, 35, 36). Curcumin, quercetin, luteolin, and apigenin have been studied for their effects on NF-*κ*B-associated inflammatory signalling, while andrographolide, curcumin, resveratrol, and baicalein have been shown to affect NLRP3 inflammasome-associated pathways (40, 41, 55). Resveratrol, quercetin, sulforaphane, and curcumin have also been linked to SIRT1- and NRF2-dependent modulation of mitochondrial and antioxidant responses (57). Hence, these findings suggest a molecular framework for additional research into phytochemicals as modulators of cardiovascular immunometabolism.

Nonetheless, pathway regulation in cell or animal models should not be regarded as proof of proven cardiovascular therapeutic effectiveness. A significant percentage of the existing information is still preclinical, and human research directly examining cardiovascular immunometabolic processes are few (3, 7, 25). Further, it is unclear whether the cardiovascular effects attributed to phytochemicals are the result of direct modulation of immune-cell metabolism or secondary changes in systemic glucose and lipid metabolism, oxidative stress, mitochondrial function, or inflammatory status. As a result, mechanistic findings should be considered as hypothesis-supporting rather than definite proof of clinical improvement. Due to the linked metabolic, oxidative, mitochondrial, and immunological pathways involved in cardiovascular inflammation, phytochemicals' polypharmacological properties may be pertinent to CVD. As a result, network pharmacology has been suggested as a paradigm for investigating multitarget activities instead of concentrating just on molecular targets (6, 58). Multitarget activity, however, may not always result in more effective treatment. Pharmacokinetic complexity, varied dose-response relationships, off-target effects, and possible herb–drug interactions can all be introduced by simultaneous effects on many pathways. Rather than just counting the number of pathways impacted, the quality, repeatability, biological relevance, and translational power of the evidence should be taken into consideration when evaluating the therapeutic value of phytochemicals.

The significant variability of phytomedicines is a substantial translational constraint. Phytochemical composition and, in turn, biological activity can be influenced by plant species, geographic origin, growth circumstances, harvesting period, extraction techniques, formulation, and manufacturing methods (7, 8). This unpredictability might make it more difficult to compare experiments and lead to conflicting experimental results. For repeatable mechanistic and clinical research, standardised botanical extracts and thoroughly characterised multi-component phytopharmaceuticals are crucial. To increase the comparability and translational utility of phytomedicine research, future investigations should be accompanied with quality control, chemical characterisation, pharmacokinetic evaluation, and uniform reporting. Pharmacokinetic constraints are yet another significant obstacle to translation. Several phytochemicals have limited tissue exposure, rapid metabolism, low water solubility, or poor oral bioavailability, which may limit the quantities that may be reached at cardiovascular sites following traditional administration. Liposomes, phytosomes, polymeric nanoparticles, and lipid-based systems are examples of nanophytomedicine techniques that may enhance stability, delivery, plasma exposure, and tissue targeting; however, before their cardiovascular applications can be established, these techniques also need to be thoroughly evaluated for safety, pharmacokinetics, formulation reproducibility, and clinical significance. Therefore, rather than being a proven remedy for the translational constraints of phytochemicals, the possibility of such delivery techniques remains a field for additional research. Additionally, current availability of evidence demonstrates a distinct discrepancy between mechanistic and clinical studies. A large portion of the data on AMPK activation, NF-*κ*B and NLRP3 suppression, macrophage modulation, mitochondrial restoration, and antioxidant signalling has been produced utilising animal models and *in vitro* systems. These studies offer valuable mechanistic information, but it is not always possible to generalise the results to human CVD. Adequately powered trials assessing major cardiovascular events or validated cardiovascular immunometabolic endpoints are less common in the existing clinical literature than surrogate outcomes or cardiometabolic risk variables (36). However, the current data does not prove that phytomedicines can replace evidence-based cardiovascular therapies, but it does encourage more research into them as possible immunometabolic modulators.

The translational difference between clinical evidence and mechanistic plausibility is further demonstrated by the comparison with immunometabolic therapies. Clinical or translational evidence that modulation of metabolic and inflammatory pathways can affect CVD processes has been provided by metformin, rapamycin, canakinumab, and sodium-glucose cotransporter-2 inhibitors (1, 48, 50, 52). Although these instances bolster the scientific justification for focusing on immunometabolism, they should not be taken as proof that phytochemicals are equally effective in the cardiovascular system. Instead, they provide a framework for further evaluating the molecular and clinical data supporting phytomedicines. Therefore, standardised plant extracts and well-defined phytopharmaceutical formulations should be given high priority for thorough evaluation of pharmacokinetics, pharmacodynamics, safety, and herb–drug interactions. When applicable, validated immunometabolic indicators should be combined with clinically significant cardiovascular outcomes in randomised controlled studies. Additionally, tissue distribution, inter-individual variability, dosage exposure, and clinically significant formulation features should all receive more consideration. Single-cell RNA sequencing, spatial transcriptomics, metabolomics, proteomics, single-cell immunometabolomics, and systems or network pharmacology are examples of advanced techniques that could be used to identify cell-specific responses and elucidate the connections between immunometabolic signalling and phytochemical exposure (32). Artificial intelligence-assisted techniques may aid in the discovery of promising chemicals and molecular targets; however, computational predictions must be validated in the laboratory and clinical validation. According to the evidence currently available, cardiovascular immunometabolism is a physiologically appropriate paradigm for examining phytomedicine-based therapies. Although there is evidence that phytochemicals can affect several interrelated inflammatory, metabolic, mitochondrial, and antioxidant pathways, much of it is still preclinical and mechanistic. Improved standardisation, pharmacokinetic and safety characterisation, repeatable formulations, suitable evaluation of herb-drug interactions, and sufficiently powered clinical studies are all necessary for their clinical translation. Therefore, before the immunometabolic effects of phytomedicines can be converted into proven cardiovascular treatment techniques, a careful, evidence-based strategy merging molecular validation with clinical outcome research is needed.

## 8 Conclusions

Cardiovascular immunometabolism offers a biologically appropriate paradigm for analysing the relationship between metabolic failure, immunological activation, oxidative stress, mitochondrial abnormalities, and cardiovascular disease. Various pathways, such as AMPK, mTOR, NF-*κ*B, NLRP3, SIRT1, and NRF2, play a role in these processes. Phytochemicals have been shown to influence these pathways in both cellular and animal models. However, the current research does not show that phytomedicine pathway modification results in clinically significant cardiovascular benefit. Major concerns remain about pharmacological exposure, bioavailability, tissue distribution, dose-response relationships, safety, herb-drug interactions, phytochemical variability, and the causative contribution of various immunometabolic pathways to cardiovascular outcomes. Human evidence is far less established than the preclinical literature, and well powered randomised controlled studies examining clinically important cardiovascular outcomes are still needed. Future studies should therefore prioritise standardised and chemically characterised preparations, thorough pharmacokinetic and pharmacodynamic evaluation, mechanistic validation in disease-relevant models, and well-designed clinical trials that include important immunometabolic biomarkers. The integration of multi-omics, systems pharmacology, single-cell techniques, nanophytomedicine, and computational methods may aid in target selection and formulation creation, but these technologies should supplement rather than replace thorough clinical validation. Thus, phytomedicines should be viewed as promising research prospects rather than established alternatives to evidence-based cardiovascular therapy.
